# Comments on “Parental assessment and management of children’s postoperative pain: observational cohort study”

**DOI:** 10.1097/MS9.0000000000003986

**Published:** 2025-10-07

**Authors:** Wei Zhong, Junhui Zhou

**Affiliations:** Department of Anesthesiology, Henan Provincial Chest Hospital (Chest Hospital of Zhengzhou University), Zhengzhou, Henan, China

*Dear Editor,*

We read with considerable interest the recent observational cohort study by Desalegn *et al*^[1]^ titled “Parental assessment and management of children’s postoperative pain,” which provides a timely examination of the knowledge and practices of Ethiopian parents in this critical aspect of pediatric care^[1]^. This commentary was prepared without the use of any artificial intelligence (AI) tools, in accordance with the TITAN (Transparency In The reporting of Artificial INtelligence) Guidelines 2025^[2]^. The authors rightly highlight a concerning disconnect between parental perceptions of pain and those of clinical staff, alongside a generally low-to-moderate level of knowledge regarding pain relief strategies. While the study documents these gaps, it also invites a deeper, more systemic critique of how postoperative pain is conceptualized and managed within family-centered care models, particularly in low-resource settings.

A primary consideration beyond the scope of the current discussion is the methodological challenge of cross-culturally measuring pain. The study employed the Parents’ Postoperative Pain Measure (PPPM) and nurse-assessed Face, Legs, Activity, Cry, Consolability/Numeric Rating Scale scales, reporting a significant discrepancy in scores^[1]^. While this difference is attributed to deficits in parental knowledge, it may also reflect fundamental divergences in pain expression, interpretation, and cultural validation. Pain is not a purely somatic experience, but is deeply embedded in cultural narratives and communicative practices^[3]^. In many non-Western contexts, expressive behaviors may be subdued or amplified based on social norms, which can lead to systematic underestimation or overestimation by healthcare providers using standardized tools developed in Western populations^[4]^. Therefore, what appears as a “gap” in assessment may partly stem from a cultural gap in pain interpretation – one that is not easily bridged by knowledge transfer alone.

Furthermore, the finding that younger parents employed more pain management strategies than older parents suggests generational or educational shifts, but it might also indicate differing levels of trust in biomedical institutions or familiarity with clinical environments. In many resource-limited settings, parents’ reluctance to administer analgesics may stem from well-founded fear of side effects, addiction, or financial burden^[5]^. Similarly, the preference for non-pharmacological strategies such as hugging, holding, and assisting with daily activities, though interpreted as a limited repertoire, may in fact represent a robust, culturally grounded approach to comfort that is not yet fully integrated into biomedical pain management protocols^[6]^.

This study’s conclusion calls for more parental education, which is undoubtedly necessary. However, educational interventions must be contextualized and co-designed with local communities to avoid reproducing top-down, potentially neocolonial approaches to pain “management.” Simply training parents to think like nurses may overlook the unique expertise that parents hold regarding their children’s behaviors and needs^[7]^. A more transformative approach would involve developing shared assessment tools that incorporate a local understanding of pain and distress, thereby facilitating more effective communication between families and clinicians.

Finally, while the study focuses on parents’ roles, it implicitly critiques systemic failure in supportive perioperative care. The fact that nearly all children were rated by parents as having significant pain (PPPM ≥6) should alert us not only to parental misperception but also to the possible systemic undertreatment of pain. In settings with high nurse-to-patient ratios and limited analgesic availability, parental involvement is not merely beneficial but essential. However, parents cannot compensate for structural shortcomings without adequate training, support, or inclusion in the clinical team.

In summary, while Desalegn *et al*^[1]^ provide valuable baseline data on parental practices, we encourage a shift in interpretation from what parents lack to what they do and from individual knowledge gaps to systemic and cultural barriers. Future research should explore culturally adapted pain communication frameworks; participatory intervention design; and the role of trust, advocacy, and shared decision-making in improving postoperative outcomes for children in global contexts.

## Data Availability

Not applicable.
